# The protease code of the bacterial gut microbiota: ecological regulation, barrier disruption, and rewiring host defense and signaling

**DOI:** 10.1080/19490976.2026.2687149

**Published:** 2026-06-10

**Authors:** Ulrich Eckhard, Juan Sebastián Ramírez-Larrota, Eva Estevan-Morió, F. Xavier Gomis-Rüth

**Affiliations:** a Department of Structural and Molecular Biology, Synthetic Structural Biology Group, Molecular Biology Institute of Barcelona (CSIC), Barcelona Science Park, Barcelona, Catalonia, Spain; b Department of Structural and Molecular Biology, Proteolysis Laboratory, Molecular Biology Institute of Barcelona (CSIC), Barcelona Science Park, Barcelona, Catalonia, Spain; c Graduate Program in Biotechnology, Faculty of Pharmacy and Food Sciences, University of Barcelona, Barcelona, Catalonia, Spain

**Keywords:** Gut microbiota, peptidases, proteolysis, host-microbe interaction, E-cadherin, virulence factors

## Abstract

The human gastrointestinal tract is home to a dense and diverse microbial ecosystem that functions as a complex superorganism. Yet, the enzymatic machinery responsible for orchestrating this coexistence remains underexplored. Among these enzymes, microbial peptidases (proteases) represent pivotal effectors of the host-microbe interface. Far from acting merely as nonspecific digestive degraders, these proteases serve as highly specific signaling enzymes that can tip the balance between eubiosis and dysbiosis, functioning as critical architects of biofilm structure while also acting as potent virulence factors. This review provides a focused analysis of the microbial proteolytic landscape. We first examine how these enzymes govern community dynamics through the remodeling of biofilm protein matrices and the quenching of peptide-based quorum sensing. At the host-microbe boundary, we detail the hierarchical dismantling of barriers: from mucinases that compromise the protective mucus layer, to the systematic cleavage of adherens junctions by key effectors including enterotoxigenic *Bacteroides fragilis* toxin, *Campylobacter jejuni* HtrA, and *Porphyromonas gingivalis* gingipains. Finally, we explore how proteases transcend physical destruction to manipulate cellular signaling, subverting mucosal immunity via immunoglobulin A cleavage and driving inflammation through the activation of protease-activated receptors.

## Graphical abstract

**Figure:**
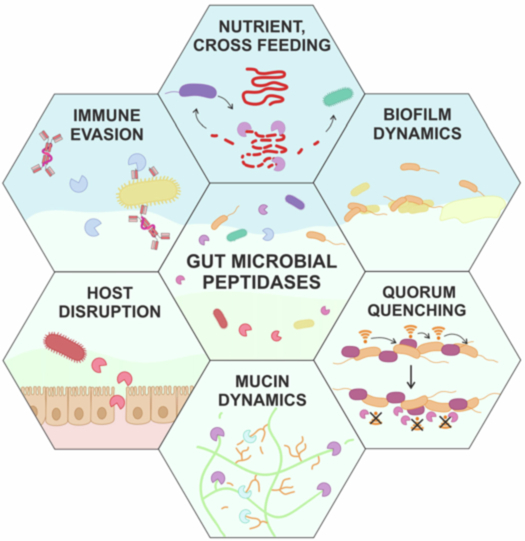

## 1. Introduction

The human gastrointestinal (GI) tract is home to a microbial ecosystem of staggering complexity and density, functioning as a metabolic organ that fundamentally extends human physiological capabilities. While historical estimates often cited a 10:1 ratio of bacterial to human cells,^1^ ^,^ ^2^ the field currently estimates the total number of bacterial cells in a “reference man” of 70 kg to be approximately 3.8 × 10^13^, a number roughly equivalent to the total human cell count of 3.0 × 10^13^.^3^ However, this revised ratio does not diminish the biological significance of the microbiota; rather, it highlights the immense metabolic potency of this biomass. This “superorganism” has co-evolved to perform essential functions that the human host cannot execute independently. The most well-characterized of these is the saccharolytic fermentation of complex dietary polysaccharides, such as starch, inulin, and pectin, into short-chain fatty acids (SCFAs) in the colon.^4^ These metabolites serve not only as the primary energy source for colonocytes by supplying 60-70% of their energy requirements,^5^ but also as critical signaling molecules that regulate mucosal immunity, maintain barrier integrity via tight junction modulation, and influence systemic metabolic homeostasis.^6-8^ Beyond fermentation, the microbiota is indispensable for the *de novo* biosynthesis of essential micronutrients, including Vitamin K (menaquinones) and water-soluble B-vitamins such as biotin (B_7_), folate (B_9_), and cobalamin (B_12_).^9^ ^,^ ^10^ Furthermore, the gut microbiota orchestrates the biotransformation of primary bile acids synthesized by the host liver into a diverse pool of secondary bile acids such as deoxycholic acid and lithocholic acid. These microbial modifications alter the hydrophobicity and signaling properties of bile acids, impacting their interaction with host nuclear receptors, thereby regulating glucose metabolism, lipid absorption, and inflammation.^11-13^

However, the molecular dialog between microbe and host is mediated not just by these small-molecule metabolites, but by a vast arsenal of catalytically active proteins, known as enzymes. While carbohydrate-active enzymes (CAZymes) have received significant attention,^14^ genomic and metagenomic analyzes indicate that protease-encoding genes constitute approximately 1–6% of the average bacterial genome.^15^ This pervasive genetic potential suggests that proteolysis is a fundamental regulatory mechanism in the gut. Yet, genomic presence does not strictly correlate with phenotypic output.^16-18^ Proteolytic activity is heavily regulated at the post-translational level; many enzymes are secreted as inactive zymogens to prevent premature degradation of bacterial cellular components, or are inhibited by host-derived antiproteases (e.g., serpins, cystatins) and microbial inhibitors upon secretion.^19^ ^,^ ^20^ Consequently, to capture the true enzymatic landscape, the field is moving beyond static metagenomics toward functional metaproteomics,^21-23^ and more specifically, activity-based protein profiling (ABPP).^24^ ^,^ ^25^ ABPP utilizes chemical probes equipped with an electrophilic “warhead” that covalently reacts solely with the catalytic active site of specific enzyme classes (e.g., fluorophosphonates for serine hydrolases), thereby distinguishing functionally active enzymes from their dormant, zymogenic, or inhibited forms.^26^ ^,^ ^27^ This approach enables high-resolution mapping of the “functional gut degradome”, distinguishing between microbial composition (taxonomy), metabolic potential (genomics), and realized enzymatic function (activity).^28^ ^,^ ^29^

**Figure 1.**
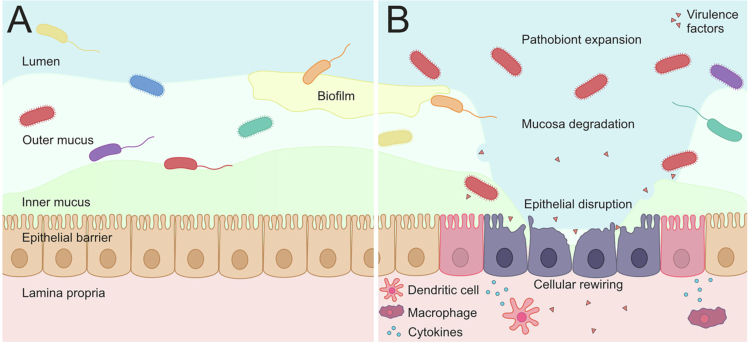
Comparative overview of the intestinal environment in health and disease. (A) Eubiosis. A stable healthy intestinal barrier is characterized by a stratified mucus layer with a dense, bacteria-free inner zone (dark green) and a loose outer habitat (light green) hosting a healthy biofilm (yellow) and a diverse commensal microbiota (multicolored rods). Below, a uniform monolayer of healthy enterocytes (tan) ensures barrier integrity.^33^ ^,^ ^70^ (B) Dysbiosis. Pathological transition is driven by the over proliferation of opportunistic or pathogenic taxa (pathobiont expansion; red bacilli), coupled with the secretion of enzymatic virulence factors (red triangles) that drive mucosa degradation and epithelial disruption. Translocation of microbial effectors leads to cellular rewiring and phenotypic changes in stressed enterocytes (pink and purple cells), triggering inflammation, immune cell recruitment (dendritic cells and macrophages; shown in pink and purple, respectively), and the release of pro-inflammatory cytokines (light blue circles).^31^ ^,^ ^36^

In the GI tract, the total proteolytic load is a complex combination of host-derived enzymes, such as pepsin (MEROPS peptidase database family A1) in the stomach, and trypsin, chymotrypsin, and elastase (family S1), and carboxypeptidases (family M14) in the small intestine, alongside mammalian matrix metalloproteinases (MMPs; family M10) involved in tissue remodeling,^84^ and those produced by the resident microbiota.^31^ ^,^ ^32^ While host proteases are temporally and spatially regulated to support digestion and tissue homeostasis, microbial peptidases are produced either constitutively or in response to environmental cues such as nutrient limitation, quorum sensing signals, or host immune effectors. These enzymes are anchored to the bacterial surface, released freely into the lumen, or embedded within the host mucus layer.^33^ Regardless of their localization, the significance of these microbial enzymes is profound. Peptidases shape the gut environment by **(i)** degrading structural scaffold proteins within the extracellular polymeric substance (EPS) during microbial biofilm remodeling^34^ or within the host extracellular matrix to drive virulence; **(ii)** activating or inactivating signaling receptors; and **(iii)** modulating or dismantling components of the immune system, thereby enabling immune evasion and creating a viable niche for both commensal and pathogenic bacteria (Figure 1).^33-40^ Crucially, this proteolytic landscape exhibits a distinct longitudinal gradient.^41^ ^,^ ^42^ The upper GI tract (stomach and small intestine) functions as a “proximal checkpoint”, defined by high concentrations of host digestive proteases, rapid transit times, bile stress, and a microbiota adapted to the processing of specific dietary antigens. In contrast, the distal colon harbors a dense, anaerobic community where proteolysis shifts functionally towards nitrogen scavenging from endogenous sources (mucins, sloughed cells) and complex interspecies signaling.^31^ ^,^ ^43-45^ This spatial segregation also dictates the functional repertoire: while upper tract enzymes often target dietary substrates (e.g., gluten degradation by *Rothia* spp.^46^ or gastric-active prolyl endopeptidases like neprosin,^47^) colonic proteases are primary drivers of ecosystem architecture, host-microbe signaling, and the subversion of mucosal immunity.^31^ ^,^ ^36^ ^,^ ^41^

An additional layer of complexity arises from microbial flux between anatomically distinct mucosal sites.^48^ The GI tract does not represent an isolated ecological unit, but rather a continuum that can be perturbed by microbial translocation from the oral cavity, particularly under conditions of altered gastric acidity,^49^ ^,^ ^50^ immunodeficiency,^51^ or aging.^52^ ^,^ ^53^ Importantly, age-dependent susceptibility is driven by the natural decline in host checkpoints. Once these defenses are breached, especially inflammophilic periodontitis-associated oral pathobionts^54^ gain a distinct competitive advantage in the dysbiotic gut,^39^ allowing them to ectopically colonize the lower GI tract and exert disproportionate effects on mucosal immunity and inflammatory tone, establishing a functional oral–gut axis mediated in part by secreted peptidases.^52^ ^,^ ^55^ Importantly, microbial proteolysis does not operate in isolation within the gut ecosystem. Proteases act in tight functional coordination with other enzymatic and physicochemical determinants, including glycoside hydrolases, bile acids, short-chain fatty acids, and mechanical forces such as peristalsis and shear stress.^43^ ^,^ ^56^ Rather than functioning as universally dominant drivers, microbial peptidases are uniquely positioned as irreversible molecular switches.^57^ Through precise peptide bond cleavage, they can permanently remodel structural barriers,^58^ ^,^ ^59^ activate or terminate signaling pathways,^60^ ^,^ ^61^ and rewire host-microbe interactions^62^ ^,^ ^63^ in ways that are not readily reversible by metabolic flux alone. It is this capacity for specificity, irreversibility, and signal amplification that distinguishes proteolysis as a critical regulatory axis within the broader, multi-layered gut ecosystem. Importantly, given the immense diversity of the bacterial gut microbiota, this review does not try to provide an exhaustive list of protease producers. Instead, we deliberately concentrate on a selected group of well-characterized species to exemplify mechanistic frameworks of broader proteolytic paradigms, but which likely extend to a wide array of gut bacteria beyond those discussed here.

This review aims to categorize and contextualize the diverse functions of gut microbial peptidases, with a special focus on the following three domains: **(1)** ecological regulation: how peptidases govern community dynamics and interspecies competition through nutrient acquisition, biofilm remodeling, and the quenching of peptide-based quorum sensing; **(2)** pathogenicity and barrier disruption: the specific mechanisms by which proteases breach barriers, spanning from mucinases that compromise the protective mucus layer to the systematic cleavage of adherens junctions (AJs) by key effectors including *Bacteroides fragilis* toxin (fragilysin) and *Campylobacter jejuni* HtrA; and **(3)** immune subversion and signaling: how proteolytic enzymes subvert host surveillance via mechanisms including IgA cleavage and manipulate physiological responses through protease-activated receptors (PARs).

## 2. Microbial peptidases as architects of gut microbiota organization and structure

The establishment of a stable niche within the highly competitive and spatially structured gut ecosystem is a prerequisite for long-term microbial persistence.^64^ In this dense environment, peptidases function as essential survival tools, orchestrating metabolic networks and driving the physical organization of the community.^65-67^ Thus, these enzymes serve as fundamental ecological regulators that dictate resource availability (via protein hydrolysis),^44^ modulate biofilm architecture (by processing EPS components such as curli fibers and adhesins),^68^ and govern interspecies communication, particularly among Gram-positive bacteria.^69^ Crucially, these activities define the equilibrium between a healthy intestinal environment (eubiosis) and the pathological transition to a pathobiont-driven state (dysbiosis). This transition is governed by a functional spectrum of microbial peptidases, ranging from metabolic “sustainers” in the healthy gut to invasive “switches” that drive barrier collapse (Figure 1).^32^ Accordingly, the examples discussed throughout this review encompass both resident gut commensals and transient or opportunistic taxa, whose proteolytic strategies, while not confined to stable colonization of the healthy gut, provide broadly applicable mechanistic insight into microbial ecology and host interaction within the GI tract.

### 2.1. Nutrient acquisition and proteolytic cross-feeding

The most fundamental role of extracellular peptidases is nutritional.^71^ While gut bacteria typically prioritize dietary glycans as their primary energy and carbon source,^72^ the distal colon is often a nitrogen-limited environment.^73^ Proteins act as a dual substrate: they are not only the essential reservoir for nitrogen biosynthesis (amino acids) but, upon carbohydrate depletion, serve as an alternative carbon source.^45^ ^,^ ^74^ ^,^ ^75^ These substrates are derived from two main nutrient pools: **(i)** dietary proteins that escape upper GI digestion, including animal-derived connective tissues like collagen and elastin, and plant-derived storage proteins such as gluten and gliadin;^76^ and **(ii)** endogenous material, such as the lubricating barrier of the gut formed by mucins and sloughed epithelial cells, facilitating nutrient recycling.^56^ ^,^ ^77^ ^,^ ^78^

#### 2.1.1. Functional division of labor: primary degraders and scavengers

Not all gut bacteria possess the same proteolytic capacity. The ecosystem relies on so-called “keystone” proteolytic species to initiate the breakdown of complex proteins.^79^ ^,^ ^80^ Recent pan-genomic analyses and metabolic modeling have refined our understanding of these functional guilds.^81^ ^,^ ^82^ While genera such as *Clostridium* (e.g., *C. histolyticum*, *C. perfringens*) and *Staphylococcus* (e.g., *S. aureus*) are classically recognized for secreting potent metalloproteases like collagenase^83^ (MEROPS^30^ family M9) and aureolysin^85^ (family M4) to dismantle high-molecular-weight substrates, members of the phylum Bacteroidota (formerly Bacteroidetes), including *Cytophaga* spp. with their complex collagenolytic enzymes,^86^ ^,^ ^87^ are arguably the most versatile proteolytic architects in the healthy adult gut.^65^ ^,^ ^71^ For instance, *Bacteroides caccae* has been identified as a proteolytic powerhouse, with single genomes encoding up to 156 different proteases.^88^ This extensive repertoire allows *B. caccae* to adapt to high-protein diets and utilize proteins as a primary energy source. However, the strategy of Bacteroides differs significantly from members of the phylum Bacillota (formerly Firmicutes). While Bacillota such as *Clostridium* and *Lactococcus* species often secrete specialized proteases, including host-protein specific enzymes like collagenases, into the lumen as “public goods”,^71^ ^,^ ^83^ ^,^ ^89^ Bacteroides species frequently utilize surface-tethered lipoproteins or sequester proteases within the periplasm.^90-93^ This strategy mirrors the “selfish” mechanisms described for glycan utilization,^94^ minimizing “cheating” by neighboring sponger scavengers, i.e., bacteria that do not produce secretory proteases but take up oligopeptides and free amino acids, and allowing for the efficient coupling of substrate degradation with transport systems.^89^ ^,^ ^95^ Despite these selfish strategies, proteolytic cross-feeding remains a cornerstone of gut ecology.^93^ ^,^ ^96^ “Keystone” degraders that do secrete extracellular enzymes generate oligopeptides within the open luminal environment. These peptides function as “public goods” that support the growth of non-proteolytic scavenger bacteria. This establishes a cooperative dynamic where distinct bacterial populations maintain nutritional interdependence.^97^ ^,^ ^98^ In exchange for the liberated nitrogen, scavengers often provide essential micronutrients, such as B-vitamins, to the proteolytic strains, or remove metabolic end-products that would otherwise inhibit the degraders.^99^ ^,^ ^100^ This phenomenon aligns with the “Black Queen Hypothesis”,^101^ where gene loss (e.g., loss of protease genes) is evolutionarily advantageous if the function is reliably provided by the community.^93^

#### 2.1.2. The Stickland reaction: the metabolic engine of proteolysis

A critical, yet often overlooked, mechanism of proteolytic energy generation in the anaerobic gut is the Stickland reaction,^102^ utilized primarily by specific proteolytic clostridia such as *Clostridium sporogenes* and *Clostridioides difficile*. This unique metabolic pathway couples the oxidation of one amino acid (the electron donor, typically branched-chain amino acids like leucine, valine, or isoleucine) to the reduction of another (the electron acceptor, typically proline or glycine).^103^ Mechanistically, this process operates through two synchronized branches. In the oxidative arm, the donor amino acid undergoes oxidative deamination and decarboxylation to yield a short-chain fatty acid derivative, such as isovalerate from leucine, while simultaneously generating ATP via substrate-level phosphorylation and releasing reducing equivalents. These equivalents (NADH or reduced ferredoxin) are subsequently consumed in the reductive arm, where the acceptor amino acid is converted into its corresponding product—such as 5-aminovalerate from proline—to restore cellular redox balance.^104^ This metabolic coupling marks *C. sporogenes* and similar organisms as “proteolytic keystones” that drive the thermodynamic flow of nitrogen fermentation. Furthermore, the resulting byproducts (e.g., isobutyrate, isovalerate, 5-aminovalerate) circulate systemically as bioactive signals modulating host immunity and metabolism.^105^ ^,^ ^106^ Thus, the Stickland reaction fundamentally links microbial bioenergetics to physiological host regulation.

### 2.2. Biofilm dynamics: the “build, maintain, and disperse” cycle

In the dynamic environment of the GI tract, the traditional view of bacteria as solitary, planktonic entities is the exception rather than the rule. Most gut bacteria persist as members of complex, surface-attached communities, illustratively often referred to as “cities of microbes”.^107^ ^,^ ^108^ The structural integrity of these communities is maintained by the EPS. While often characterized primarily by its polysaccharide content, the EPS scaffold is critically reinforced by extracellular DNA (eDNA)^109^ and a meshwork of structural proteins, particularly amyloid-like fibers.^110-112^ Within this context, microbial peptidases play a versatile role,^113^ acting as indispensable constructors during biofilm assembly,^114^ ^,^ ^115^ architects during structural remodeling,^67^ and essential effectors of dispersal.^116^ ^,^ ^117^ This reflects the fundamental biological principle that biofilms are not static structures but dynamic systems undergoing a continuous cycle of growth, maturation, detachment, and regeneration.

#### 2.2.1. Matrix assembly and surface anchoring

The biofilm assembly phase relies on precise, enzyme-mediated proteolytic processing events. A prime example is the activity of sortases, a family of cysteine transpeptidases that cleave a peptide bond within a donor substrate and transfer the carboxyl group to an amino acceptor. These enzymes recognize a conserved C-terminal sorting signal, typically the “LPXTG motif”, on secreted surface proteins.^118^ ^,^ ^119^ Upon recognition, sortase cleaves the peptide bond between the threonine and glycine residues of the sorting signal, forming a thioacyl enzyme–substrate intermediate. Crucially, the subsequent transfer step defines the architectural outcome. Housekeeping sortases (Class A; family C60) covalently attach the threonine carboxyl group to the free amino group of the peptidoglycan cross-bridge, thereby anchoring proteins to the cell wall meshwork of N-acetylglucosamine (NAG) and N-acetylmuramic acid (NAM) units. In contrast, pilin-specific sortases (Class C; family C60) of Gram-positive bacteria typically transfer the substrate to the *ε*-amino group of a lysine residue on an adjacent pilin subunit, driving pilus polymerization.^120^ Both mechanisms are vital for the surface display of adhesins and surface proteins such as microbial surface components recognizing adhesive matrix molecules (MSCRAMMs), which collectively mediate initial attachment to host tissues or co-aggregation with other microbes.^121^ Flagella also contribute to the initial attachment phase, though their fate varies by species. In some organisms (e.g., *Vibrio* spp.), flagellar motility promotes early adherence and is subsequently downregulated as cells transition to a sessile state, whereas in others (e.g., *Escherichia coli*), flagella facilitate surface approach and microcolonial attachment, and may become physically entangled within the developing matrix.^122-124^

#### 2.2.2. Maturation and structural remodeling: functional amyloids

Between the initial assembly and final dispersal, the biofilm undergoes a phase of maturation and remodeling. During this stage, mechanical stability increasingly relies on the polymerization of functional amyloids, such as curli fibers in Enterobacteriaceae (e.g., *E. coli* and *Salmonella enterica*) or TasA fibers in *Bacillus subtilis*.^111^ ^,^ ^125^ Unlike pathological amyloids associated with human neurodegenerative diseases,^126^ these bacterial amyloids are functional biomaterials produced via tightly regulated biosynthetic pathways to avoid cellular toxicity. The formation of these structures is strictly regulated by proteolysis and accessory factors.^127^ For curli, the major subunit CsgA is secreted as an unstructured, soluble monomer, whose polymerization into amyloid fibers is nucleated at the cell surface by the minor subunit CsgB. To prevent premature and potentially lethal polymerization within the periplasm or cytoplasm, chaperones such as CsgC actively inhibit CsgA aggregation prior to secretion.^128^ More generally, proteolytic removal of N-terminal inhibitory sequences from amyloid subunits prevents premature intracellular aggregation and ensures that assembly into fibers occurs only after secretion into the extracellular space. Once assembled, these mechanically robust, tightly packed cross-*β*-sheet quaternary structures confer the biofilm with exceptional resistance against host digestive enzymes, chemical stressors, and physical shear forces, thereby creating a durable protective matrix.^111^ ^,^ ^112^ Concurrently, proteolytic activity continuously sculpts the biofilm architecture during maturation. For example, in *Pseudomonas* spp., the periplasmic protease LapG (family C55) cleaves the large surface adhesin LapA once irreversible attachment is no longer advantageous, enabling architectural remodeling or detachment.^129^ ^,^ ^130^ Proteases also contribute to the formation of channels within the dense EPS, facilitating nutrient and oxygen diffusion to deeper layers of the biofilm.^110^ ^,^ ^117^ Moreover, peptidases act in concert with DNases, which cleave extracellular DNA, and glycoside hydrolases, which remodel polysaccharide components, to dynamically adjust EPS composition in response to population growth and environmental change.^109^ ^,^ ^131^ This enzymatic “maintenance” preserves structural integrity while providing the plasticity required for long-term persistence in the gut ecosystem.

#### 2.2.3. Active dispersal and niche expansion

Under conditions of nutrient starvation, waste accumulation, or high population density, bacteria initiate an active escape strategy known as dispersal.^132^ This involves the upregulation of secreted proteases designed to hydrolyze the adhesive peptide bonds holding the community together. For instance, *Enterococcus faecalis* secretes the potent gelatinase GelE, a metalloprotease (family M4) that cleaves bacterial surface adhesins and degrades host-derived fibrin clots, which are frequently incorporated as scaffolding elements into the biofilm matrix, thereby facilitating biofilm detachment.^133^ ^,^ ^134^ Similarly, *S. aureus* utilizes the metalloprotease aureolysin,^85^ a structural and functional homolog of GelE, to degrade its own biofilm proteins, most prominently the clumping factor B (ClfB).^135^ This controlled detachment allows bacteria to revert temporarily to a planktonic state or to release small clusters of cells, a process often termed “seeding dispersal”, which enables recolonization of new, nutrient-rich niches and thereby ensures the spatial spread, ecological resilience, and long-term persistence of the microbiota during environmental perturbations.^34^ ^,^ ^136^

### 2.3. Interspecies communication: quorum sensing and signal interference

In the densely populated gut ecosystem, survival depends on a delicate balance between cooperation and competition. To persist, bacteria must efficiently coordinate group behaviors with conspecifics while simultaneously securing resources against rivals. This coordination is achieved through quorum sensing (QS), a density-dependent communication mechanism that regulates critical processes such as biofilm formation, virulence factor secretion, bacteriocin production, and metabolic coordination. This molecular dialog occurs both within single-species individuals (intraspecies) and across diverse populations (interspecies).^137^ ^,^ ^138^ However, the “language” of QS is strongly shaped by the bacterial cell envelope architecture. Many Gram-negative bacteria utilize small, lipophilic molecules known as acyl-homoserine lactones (AHLs), such as N-3-oxo-dodecanoyl-L-homoserine lactone. Owing to their amphipathic nature, these signals are synthesized in the cytosol and can freely diffuse across the inner and outer membranes along a concentration gradient to bind intracellular receptors in neighboring cells.^139^ ^,^ ^140^ By contrast, Gram-positive species, which constitute the majority of the distal gut microbiota, including members of the phyla Bacillota (formerly Firmicutes), including *Clostridium* and *Lactobacillus*, and Actinobacteriota (formerly Actinobacteria) such as *Bifidobacterium*, rely primarily on autoinducing peptides (AIPs). These signals are short oligopeptides, typically 5–20 amino acids in length, that cannot passively diffuse across the thick cell-wall peptidoglycan and therefore require dedicated export, processing, and sensing machineries.^141^ ^,^ ^142^ Consequently, peptidases are integral to the entire lifecycle of the Gram-positive QS language, functioning not only as the biosynthetic machinery required to generate mature AIPs from larger precursor proteins, but also as potent effectors of quorum quenching (QQ) that degrade or inactivate these signals. In this sense, peptidases act both as the “pen” that writes the communal message and the “eraser” that silences it, enabling dynamic regulation of social behaviors in response to environmental conditions.^142-144^

#### 2.3.1. Signal generation and processing

Unlike AHLs, which are synthesized by cytosolic synthases, AIPs are encoded as gene products. They are initially synthesized as large, inactive precursor proteins consisting of an N-terminal leader peptide, the core signaling sequence, and a C-terminal tail. The transformation of this precursor into an active signal is a strictly proteolytic and spatially controlled event. This processing is typically mediated by specialized membrane-embedded transporters with intrinsic peptidase activity, such as AgrB (family C75) in *Staphylococcus* or ComA/ComB-like systems (family C39) in *Bacillus*.^141^ ^,^ ^142^ In the well-characterized *agr* (accessory gene regulator) system, AgrB acts as an endopeptidase that performs a dual catalytic function:^145^ it cleaves the C-terminal tail of the precursor peptide and facilitates the formation of a thiolactone ring between the C-terminal carboxyl group and a conserved internal cysteine side chain. Concurrently, the N-terminal leader sequence is cleaved during translocation across the membrane by a type-I signal peptidase such as SpsB (family S26).^146^ This macrocyclic architecture is critical, as it constrains the peptide into a conformation competent for high-affinity receptor binding while simultaneously protecting the C-terminus from degradation by extracellular carboxypeptidases. Once released into the extracellular milieu, mature AIPs serve as the diffusible “broadcast” signal. Because AIPs are impermeable to the bacterial cell membrane, they are detected by two-component signal transduction systems (TCSTS).^147^ These systems employ a dedicated transmembrane sensor histidine kinase that binds the peptide ligand extracellularly. Ligand engagement induces kinase dimerization and autophosphorylation, followed by phosphotransfer to a cognate cytosolic response regulator, thereby reprogramming gene expression.^148^ Without this precise proteolytic maturation and export process, the bacterium remains effectively “mute,” unable to convey population density information to conspecifics or coordinate collective behaviors such as biofilm formation, virulence factor secretion, or motility.^133^ ^,^ ^141^ ^,^ ^142^

#### 2.3.2. Quorum quenching as interference competition

However, in the highly competitive environment of the gut, these exposed communication lines are vulnerable to interception. QQ represents a sophisticated form of interference competition, in which bacteria secrete extracellular enzymes to degrade the signaling molecules of their neighbors.^143^ ^,^ ^149^ Just as some bacteria produce lactonases to degrade signaling AHLs of Gram-negative competitors,^150^ many species secrete extracellular peptidases specifically designed to degrade AIPs of Gram-positive rivals.^143^ ^,^ ^151^ This targeted proteolysis represents a non-lethal but highly effective strategy of competitive suppression. By hydrolyzing a competitor’s signal molecule, a bacterium can disrupt QS pathways and thereby prevent the neighboring population from transitioning into a coordinated state, such as initiating biofilm maturation or releasing toxins, which effectively “silences” the potential threat rather than eliminating it outright. For example, *Bacillus* species like *B. cereus* and *B. subtilis* secrete broad-spectrum proteases and interfering lipopeptides, capable of degrading peptide-based QS signals, most notably the autoinducing peptides of *S. aureus*, and likely those of other Gram-positive pathogens such as *C. perfringens*, thereby attenuating virulence and collective behaviors without directly killing the cell.^151-153^ Analogously, many organisms produce AHL-lactonases (e.g., AiiA-type enzymes) against Gram-negative bacteria, which hydrolyze the homoserine lactone ring and renders the signal biologically inactive. This prevents the signal accumulation required for quorum activation.^150^ ^,^ ^154^ Importantly, this mechanism is not solely antagonistic. In a healthy gut ecosystem (eubiosis), QQ activity may be essential to dampen signaling noise, preventing hyper-activation of virulence programs and helping to maintain community-level homeostasis. By disarming rather than destroying competitors, QQ imposes reduced selective pressure for resistance compared with conventional antibiotics. Consequently, the strategic modulation of microbial communication networks—using either engineered microbial consortia or purified quenching enzymes—represents a promising avenue for anti-virulence therapeutic development aimed at alleviating disease while preserving intestinal ecological balance.^155-157^ Nevertheless, while these findings highlight QQ as an attractive anti-virulence strategy, it is important to recognize that much of the supporting evidence derives from *in vitro* systems or non-gut infection models.^143^ Accordingly, the therapeutic exploitation of protease-mediated signal interference within the intestinal ecosystem should currently be regarded as conceptual and emerging, pending validation in physiologically relevant *in vivo* gut models.

## 3. Peptidases as key factors in pathogenicity and host barrier disruption

While the majority of the gut proteolytic repertoire serves homeostatic and digestive functions,^32^ ^,^ ^71^ a specialized subset of microbial proteases functions as potent virulence factors,^31^ ^,^ ^58^ often acting in concert with toxins^158^ and adhesins.^115^ These enzymes enable both pathogens and pathobionts, commensal members of the microbiota with pathogenic potential under conditions of dysbiosis, to breach the three primary lines of intestinal defense: **(i)** the mucus barrier, **(ii)** the epithelial monolayer, and **(iii)** the mucosal immune system.^33^ ^,^ ^159^ ^,^ ^160^ Crucially, proteolytic attack typically follows a defined spatial trajectory, initiating at the mucus-coated apical surface of the intestinal epithelium and progressing toward the basolateral compartment, the lamina propria. Initial degradation of secreted and membrane-associated mucins compromises the physical separation between luminal bacteria and the epithelial surface, increasing microbial access to epithelial receptors and junctional complexes.^161^ ^,^ ^162^ Subsequent cleavage of epithelial junctional proteins and extracellular matrix components further weakens barrier integrity, facilitating bacterial invasion and, in severe cases, translocation into the normally sterile submucosa and systemic circulation.^163^ ^,^ ^164^ Concurrently, secreted microbial proteases directly target components of the mucosal immune system. Several enteric and opportunistic pathogens produce immunoglobulin A (IgA) proteases capable of cleaving secretory IgA (sIgA) within the gut lumen, thereby impairing immune exclusion and promoting persistent colonization.^159^ ^,^ ^165^ In addition, bacterial proteases can degrade or functionally inactivate pro-inflammatory cytokines and innate immune mediators, including IL-1β and IL-6, blunting host inflammatory responses and facilitating pathogen survival within intestinal tissues.^36^ ^,^ ^166^

### 3.1. Homeostatic turnover vs. pathogenic breach

**Figure 2.**
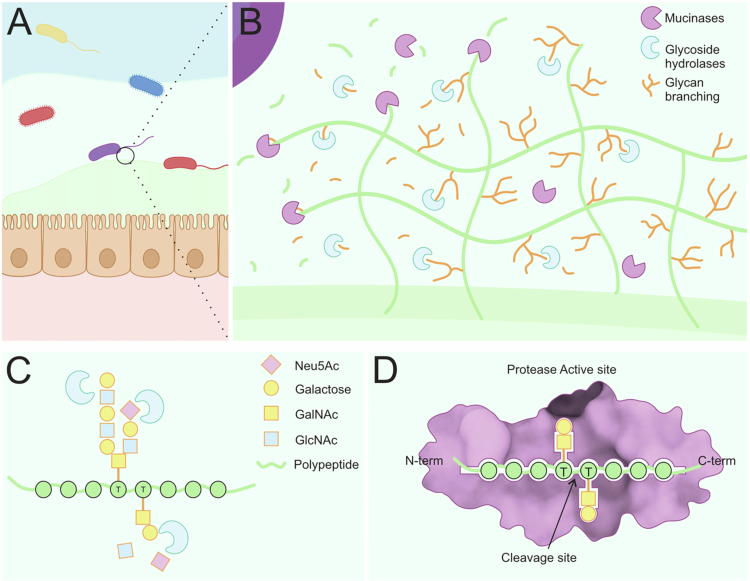
Molecular mechanisms of bacterial mucin remodeling. (A) Spatial organization of the intestinal barrier. Epithelial cell layer (beige) shielded by a stratified mucus system consisting of a dense, adherent inner layer (dark green), devoid of bacteria under homeostatic conditions, and a loose outer habitat (light green) hosting commensal microbiota adjacent to the lumen (blue).^70^ (B) Multistep enzymatic attack on the mucin matrix. Coordinated activity of glycoside hydrolases (light blue) and mucinases (purple) targeting the protective O-glycan shield (orange) and the cross-linked polypeptide scaffold (green), respectively. (C) Sequential glycan stripping. Specialized glycoside hydrolases systematically removing terminal residues (e.g., Neu5Ac, Galactose, GlcNAc), revealing underlying peptide epitopes.^43^ (D) Targeted recognition and cleavage by the *Akkermansia muciniphila* mucinase AM0627 (family M60). Site-specific proteolytic activity toward O-glycosylated peptide patches via specialized glycan-binding pockets surrounding the cleavage site. Notably, glycosylation of the threonine residue at the scissile bond is required for substrate recognition and cleavage. While mucinases can recognize diverse O-glycan profiles, the model reflects the experimentally determined complex from Protein Data Bank (PDB) entry 7YX8.^176^

The intestinal epithelium is shielded by a stratified mucus layer. While the polymeric mucin MUC5AC dominates the stomach, the intestinal barrier is primarily composed of MUC2, a large gel-forming glycoprotein.^70^ ^,^ ^167^ ^,^ ^168^ MUC2 forms a dense, hydrated mesh that functions as a physical exclusion zone, preventing direct contact between luminal bacteria and the host epithelium. Importantly, in the colon this organization is biphasic, consisting of an outer, loose layer that serves as a habitat for commensal microorganisms and an inner, firmly adherent layer that is largely sterile (Figure 2A).^70^ The structural integrity of this barrier critically relies on the molecular architecture of its principal building block. MUC2 consists of a central, heavily O-glycosylated domain that adopts a characteristic “bottle-brush” structure conferring exceptional resistance to proteolysis,^169^ flanked by non-glycosylated but cysteine-rich N- and C-terminal domains. These terminal regions are essential for intermolecular disulfide bonding, driving the polymerization of MUC2 monomers into higher-order macromolecular sheets and gels.^170^ Consequently, access to the underlying epithelium requires a coordinated, multistep enzymatic attack capable of dismantling both the protective glycan shield and the cross-linked protein scaffold of the mucin network (Figure 2B).^43^ ^,^ ^168^ ^,^ ^171^

#### 3.1.1. Physiological mucin turnover: the role of commensal proteases

It is critical to distinguish between homeostatic mucin “foraging” and pathogenic mucin “breaching,” as mucin degradation *per se* is not inherently virulent.^43^ ^,^ ^172^ On the contrary, physiological turnover is required to prevent the accumulation of structurally compromised mucus and to facilitate nutrient recycling within the gut ecosystem. This process is exemplified by *Akkermansia muciniphila*, a keystone commensal that resides within the outer mucus layer and utilizes MUC2 as a primary carbon and nitrogen source.^173^ ^,^ ^174^ *A. muciniphila* employs a controlled “grazing” strategy, deploying an extensive repertoire of glycoside hydrolases and sulfatases to remove terminal glycans (Figure 2C), followed by selective proteolysis of the exposed mucin backbone.^43^ ^,^ ^175^ ^,^ ^176^ A key effector in this process is Amuc_1434 (family A1), a secreted aspartic peptidase that efficiently cleaves the MUC2 core protein following glycan removal (Figure 2D).^174^ ^,^ ^175^ Crucially, this proteolytic activity is embedded within a beneficial metabolic feedback loop. Anaerobic mucin fermentation by *A. muciniphila* generates short-chain fatty acids, notably acetate and propionate, which stimulate host goblet cells to upregulate MUC2 gene expression and mucus secretion.^177^ ^,^ ^178^ As a result, commensal mucin degradation is coupled to barrier renewal rather than erosion. This stands in marked contrast to pathogenic strategies, which employ similar enzymatic tools but lack the reciprocal metabolic crosstalk required to promote repair, ultimately leading to net loss of barrier integrity.

#### 3.1.2. Pathogenic breaching: the mucinases

In contrast to commensal turnover, pathogens deploy mucinases as invasive effectors designed to induce rapid barrier collapse.^162^ ^,^ ^168^ Glycoside hydrolases first trim protective glycans, exposing the underlying peptide backbone.^43^ Concurrently, specialized proteases preferentially act on the non-glycosylated, cysteine-rich terminal domains of MUC2, disrupting the disulfide-mediated intermolecular cross-links that are essential for polymer network stability.^169^ ^,^ ^179^ Under physiological conditions, MUC2 polymerization is driven by covalent disulfide bonding within these terminal domains, analogous to the assembly mechanisms of other gel-forming mucins such as MUC5AC and the structurally related von-Willebrand factor.^180^ Pathogen-induced interference with this architecture results in selective depolymerization of the mucin network, compromising mucus viscoelasticity without requiring complete proteolytic digestion and effectively converting the barrier from a structured protective matrix into a more permeable, fluid layer.^162^ ^,^ ^168^ ^,^ ^181^

*Vibrio cholerae* serves as a classic model for this mechanism. The pathogen secretes the zinc metalloprotease hemagglutinin/protease (HapA),^182^ a member of the M4 family, which acts in concert with a secreted neuraminidase to expose proteolytic target sites.^181^ ^,^ ^183^ The resulting reduction in mucus viscoelasticity facilitates bacterial motility, enabling penetration of the inner mucus layer and delivery of cholera toxin directly to the epithelial surface.^184^ This mucinolytic strategy is conserved among multiple enteric pathogens. Pathogenic lineages of *E. coli*, including enteroaggregative (EAEC) and enterotoxigenic (ETEC) strains, secrete serine protease autotransporters of enterobacteriaceae (SPATEs) such as Pic and EatA.^185^ ^,^ ^186^ These high-molecular-weight serine proteases belong to the S6 family and share striking structural homology with IgA1-cleaving proteases.^187^ ^,^ ^188^ As other type-V secretion system autotransporters,^189^ SPATEs mediate their own export across the outer membrane via a C-terminal *β*-barrel pore, releasing the N-terminal protease domain into the extracellular milieu. Once secreted, these enzymes efficiently cleave mucins and mucus-associated host defense proteins,^186^ ^,^ ^187^ locally converting the mucus barrier into a gateway for epithelial access. Importantly, this proteolytic remodeling benefits not only the primary pathogen but also facilitates invasion by secondary bystander pathogens and opportunistic pathobionts.^56^ ^,^ ^190^

#### 3.1.3. Reinforcing the shield: host lectins as functional antagonists to mucinases

To counteract enzymatic fluidization, the host produces the Th2-inducible lectin intelectin-2 (Itln2), which reinforces the physical stability of the mucus matrix as a functional counterbalance to microbial mucinases.^191^ While pathogens deploy glycoside hydrolases and peptidases to strip and dismantle the MUC2 scaffold, Itln2 recognizes epitopes that only become accessible after initial bacterial deglycosylation (*i.e.*, terminal *β*-D-galactopyranose). By acting as a “molecular glue” that cross-links host mucins and tethers microbial cells to the meshwork, this lectin reinforces the barrier and neutralizes the gateways created by bacterial proteases. Moreover, Itln2 possesses broad-spectrum antimicrobial activity. By cross-linking surface glycans, it agglutinates various pathogens, including pathobionts like *S. aureus* and *K. pneumoniae*, as well as helminths and yeasts, inducing a loss of cell integrity and suppressing viability.^192^

### 3.2. Disruption of the intestinal barrier: the cadherin connection

**Figure 3.**
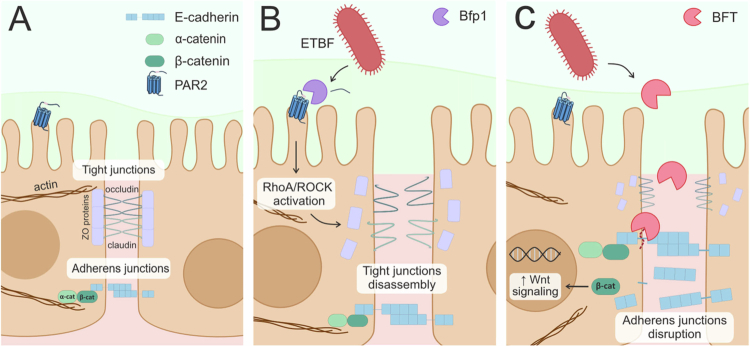
Hierarchical disruption of the intestinal epithelium by *B. fragilis* proteases. (A) Homeostatic junctional architecture. Intact apical junctional complexes comprising tight junctions (TJs) and adherens junctions (AJs). TJs are located at the apical-most part of the intercellular seal and are composed of occludin (dark blue) and claudins (teal) that seal the paracellular space (*i.e.*, the intercellular region between adjacent cells) via zonula occludens (ZO; light purple) scaffolding proteins.^194^ AJs are anchored by E-cadherin (light blue), a transmembrane protein forming homophilic interactions with neighboring cells.^196^ Intracellularly, E-cadherin is linked to the actin cytoskeleton via a catenin complex comprising *α*-catenin (light green) and *β*-catenin (dark green). (B) Bfp1-mediated apical breach. Upon colonization of the apical surface, enterotoxigenic *B. fragilis* (ETBF) secretes the serine protease Bfp1 (purple Pacman) to activate PAR2.^61^ This triggers intracellular RhoA/ROCK signaling, leading to the endocytic internalization and disassembly of TJs.^215^ This apical opening establishes a hierarchical synergy by facilitating the transepithelial access of the zinc metalloprotease *B. fragilis* toxin (BFT) to its targets at the underlying AJs. Alternatively, BFT may bypass the apical seal by exploiting transient tight junction derangement at natural cell extrusion zones, as described for *Listeria monocytogenes*,^216^ or capitalize on paracellular gateways opened by the proteases of co-colonizing microbes, such as *C. jejuni* HtrA.^217^ (C) BFT-mediated disruption and signaling hijacking. ETBF secretes BFT (red Pacman) to catalyze the ectodomain shedding of E-cadherin.^58^ ^,^ ^200^ This primary cut causes the immediate dissolution of AJs and loss of barrier integrity. Furthermore, this extracellular cleavage destabilizes the remaining intracellular domain and triggers secondary host-mediated *γ*-secretase processing,^211^ ^,^ ^212^ resulting in the liberation of *β*-catenin. This process hijacks a natural growth-sensing pathway by allowing *β*-catenin to translocate to the nucleus, where it complexes with TCF/LEF to drive aberrant Wnt signaling and the expression of proto-oncogenes such as *Cyclin D1* and *c-Myc* associated with oncogenic transformation.^213^ ^,^ ^214^ Note: panels **(B)** and **(C)** represent slightly magnified views of the intercellular space shown in **(A)** to detail molecular interactions.

Upon penetrating the mucus layer, pathogens encounter the intestinal epithelium, a single-cell-thick barrier whose integrity is maintained by apical junctional complexes (AJCs; Figure 3A).^163^ These multiprotein assemblies comprise AJs, which are anchored by E-cadherin and provide mechanical cohesion, and tight junctions (TJs), which are composed of claudins and occludin and seal the paracellular space.^193^ ^,^ ^194^ Together, these structures strictly regulate paracellular permeability, forcing selective transport of nutrients through transcellular pathways.^195^ The central molecular anchor of the AJ is E-cadherin, a calcium-dependent transmembrane protein that forms homophilic interactions between neighboring cells. Proteolytic cleavage of E-cadherin is therefore a recurring virulence strategy, capable of precipitating barrier collapse and enabling tissue invasion.^196^ ^,^ ^197^

#### 3.2.1. Fragilysin: precision cleavage and the oncogenic shift

Enterotoxigenic *B. fragilis* (ETBF) is a pathobiont strongly associated with inflammatory bowel disease and colorectal cancer.^198^ ^,^ ^199^ Its primary virulence factor, chosen here to represent E-cadherin-targeted barrier disruption, is the *B. fragilis* toxin (BFT), also known as fragilysin, a secreted zinc metalloprotease belonging to the M10 family.^188^ ^,^ ^200-202^ BFT is released by bacteria colonizing the apical mucosal surface; while it targets the underlying adherens junctions and catalyzes the ectodomain shedding of E-cadherin,^58^ ^,^ ^198^ its transepithelial access is significantly enhanced by the prior disruption of the apical tight junction seal by other co-secreted effectors, such as the serine peptidase Bfp1 (Figure 3B; see Section 3.2.2).^61^ While early models viewed BFT-mediated E-cadherin cleavage as a simple proteolytic “shedding” event, subsequent work has revealed that BFT initiates a coordinated hijacking of host junctional and signaling machinery. Although the precise epithelial receptor remained elusive for decades,^203^ recent evidence has identified the tight junction protein claudin-4 as the specific cell-surface receptor for BFT.^204^ While claudin-4 and E-cadherin are classically sequestered into distinct junctional complexes, BFT is proposed to exploit non-junctional, mobile pools of these proteins. In this model, claudin-4 functions as a critical “landing pad” that recruits BFT to the plasma membrane, properly positioning the enzyme to access and cleave the membrane-proximal ectodomain of E-cadherin via lateral diffusion. This cleavage initiates two temporally and mechanistically distinct pathological cascades. First, dissolution of AJs leads to an immediate loss of epithelial barrier integrity (Figure 3C), resulting in increased paracellular permeability and uncontrolled fluid secretion, a phenotype clinically manifested as inflammatory diarrhea.^198^ From an ecological perspective, this acute barrier disruption likely benefits the bacterium by displacing competing commensals and releasing nutrient-rich inflammatory exudates.^205^ ^,^ ^206^ Histologically, the compromised epithelial seal permits the translocation of luminal antigens, including lipopolysaccharide, into the lamina propria and submucosa, driving the recruitment of innate immune cells, particularly neutrophils, and establishing an acute inflammatory state (colitis).^163^ ^,^ ^207^ ^,^ ^208^

Second, and as a delayed consequence, BFT induces a profound rewiring of host cell signaling that predisposes the tissue to oncogenic transformation.^199^ ^,^ ^208^ Under homeostatic conditions, the intracellular domain of E-cadherin sequesters *β*-catenin at the plasma membrane, preventing its nuclear signaling activity.^209^ ^,^ ^210^ BFT-mediated ectodomain shedding disrupts this restraint and, critically, generates a membrane-tethered C-terminal fragment of E-cadherin that serves as a substrate for presenilin-1 (family A22), the host *γ*-secretase complex.^211^ ^,^ ^212^ This secondary, host-encoded intramembrane cleavage represents the pivotal oncogenic step. Subsequent processing releases the E-cadherin intracellular domain and liberates *β*-catenin into the cytosolic pool (Figure 3C).^213^ Freed *β*-catenin translocates to the nucleus, where it complexes with T-cell factor/lymphoid enhancer factor (TCF/LEF) transcription factors to aberrantly activate the Wnt signaling pathway.^210^ This program, normally restricted to stem cell maintenance and tissue regeneration, drives the expression of proto-oncogenes such as c-Myc and Cyclin D1, resulting in uncontrolled epithelial proliferation.^214^ Importantly, the inflammatory phase precedes and facilitates this oncogenic shift, providing a permissive microenvironment rich in cytokines and growth factors.^208^

Thus, BFT functions not merely as a degradative junctional protease, but as a signaling agonist that exploits host *γ*-secretase–dependent processing to convert junctional disruption into oncogenic Wnt activation. This mechanism provides a direct molecular link between a microbial protease and the oncogenic transformation of the colonic epithelium (Figure 3C). However, from an evolutionary perspective, while the immediate induction of inflammation releases nutrients that transiently benefit the bacterium, the long-term consequence of carcinogenesis offers no apparent selective advantage for the microbe.^198^ ^,^ ^199^

#### 3.2.2. Bfp1: from PAR2-mediated pain and inflammation to barrier breach

A major development in 2025 has been the identification of a second bioactive protease from *B. fragilis*, designated Bfp1, based on ABPP and proteolytic screens.^61^ In contrast to ETBF-restricted BFT, Bfp1 is broadly conserved across all *B. fragilis* lineages examined to date. It is a serine protease (family S41) functioning through the specific cleavage and activation of PAR2.^61^ Leveraging ABPP with covalent serine hydrolase inhibitors, researchers isolated Bfp1 and demonstrated that its cleavage of the PAR2 N-terminus unmasks a tethered ligand, resulting in receptor activation and sensitization of colonic nociceptors.^61^ In murine models, *B. fragilis* strains lacking Bfp1 (*Δbfp1*) failed to induce the visceral pain and barrier disruption observed with wild-type infection, despite maintaining normal colonization levels. This identifies Bfp1 as a critical virulence factor that directly modulates sensory perception and neurogenic inflammation. Importantly, this mechanism aligns with independent evidence demonstrating that protease-dependent PAR2 activation sustains persistent nociceptor hyperexcitability and visceral pain in experimental models of functional GI disorders, including irritable bowel syndrome.^218^ Furthermore, this work suggests that Bfp1-mediated PAR2 activation may represent a primary driver of abdominal pain in conditions where overt tissue damage is minimal, establishing a novel pathogenic axis that is mechanistically distinct from the oncogenic pathway driven by BFT.^61^

Importantly, Bfp1-mediated activation of PAR2 also compromises structural barrier integrity.^61^ Upon proteolytic activation, PAR2 triggers intracellular signaling cascades, prominently recruiting the RhoA/ROCK (Rho-associated protein kinase) pathway via downstream G-protein signaling.^219^ ^,^ ^220^) This induces cytoskeletal rearrangement and actomyosin contraction, exerting direct mechanical tension on the apical junctional complex. This physical strain forces the endocytic internalization and redistribution of key TJ proteins, most notably zonula occludens-1 (ZO-1) and occludin, dismantling the apical seal and increasing paracellular permeability.^215^ By opening this apical gateway, Bfp1 facilitates the transepithelial access of BFT to its targets at the underlying AJs, establishing a hierarchical synergy between these two secreted *B. fragilis* proteases (Figure 3). However, Bfp1-mediated cleavage may not be the exclusive route for BFT translocation. In a highly dynamic and polymicrobial gut environment, BFT could also bypass the apical seal by infiltrating transient breaches at natural epithelial cell extrusion sites, an entry strategy well-documented for *Listeria monocytogenes*.^216^ Alternatively, BFT could capitalize on paracellular “bystander” corridors opened by the proteolytic activity of co-colonizing pathogens, such as the broadly active protease HtrA from *C. jejuni*.^217^ ^,^ ^221^

#### 3.2.3. HtrA: broad junctional degradation and paracellular invasion

While the gastric pathogen *Helicobacter pylori* provided the foundational paradigm for HtrA-mediated junctional cleavage,^222^ the homologous protease from the intestinal pathogen *C. jejuni* was selected as a representative example to illustrate the profound ecological consequences of this mechanism in the lower GI tract.^217^ ^,^ ^223^ *C. jejuni* expresses HtrA (from “high temperature requirement A”), a serine protease belonging to the S1 family. Similar to BFT, HtrA cleaves the extracellular domain of E-cadherin, thereby opening the intercellular seal. However, a critical distinction lies in the spatial and functional outcome: whereas BFT acts as a soluble toxin that projects signals across the epithelial barrier, HtrA functions as a localized breaching tool. Paradoxically, although HtrA is abundantly secreted and shed in outer membrane vesicles (OMVs), recent evidence reveals that its junction-cleaving activity is entirely restricted to the active, surface-bound fraction upon direct bacterial contact.^224^ This spatially confined proteolysis facilitates the physical entry of the bacterium into the paracellular space.^223^ ^,^ ^225^ In contrast to the highly specific cleavage of E-cadherin by BFT, *C. jejuni* HtrA exhibits broad substrate specificity within the AJC. Beyond E-cadherin, HtrA systematically cleaves multiple tight junction proteins, including occludin^226^ and claudin-8.^221^ By dismantling both AJs and TJs of the epithelial barrier, HtrA opens and maintains a permissive paracellular route, allowing the pathogen to transmigrate across the intestinal epithelium to access basolateral surfaces and establish infection.^223^ ^,^ ^225-227^ Crucially, the impact of this proteolytic activity extends beyond the primary pathogen. Recent evidence demonstrates that the local opening of cellular junctions by *C. jejuni* HtrA acts as a paracellular gateway, triggering the opportunistic transepithelial transmigration of otherwise non-invasive members of the commensal microbiota.^217^ ^,^ ^228^ This HtrA-dependent “bystander” translocation drives aberrant immune activation and represents a critical mechanism in the pathogenesis of intestinal inflammation^227-229^ and inflammatory bowel diseases.^230^ By providing a paracellular corridor for the broader microbial community, *C. jejuni* HtrA highlights how a single microbial protease can exert disproportionate ecosystem-wide effects, illustrating how proteases dictate pathological community dynamics at the mucosal barrier.^229^

### 3.3. Immune evasion: the IgA peptidases

**Figure 4.**
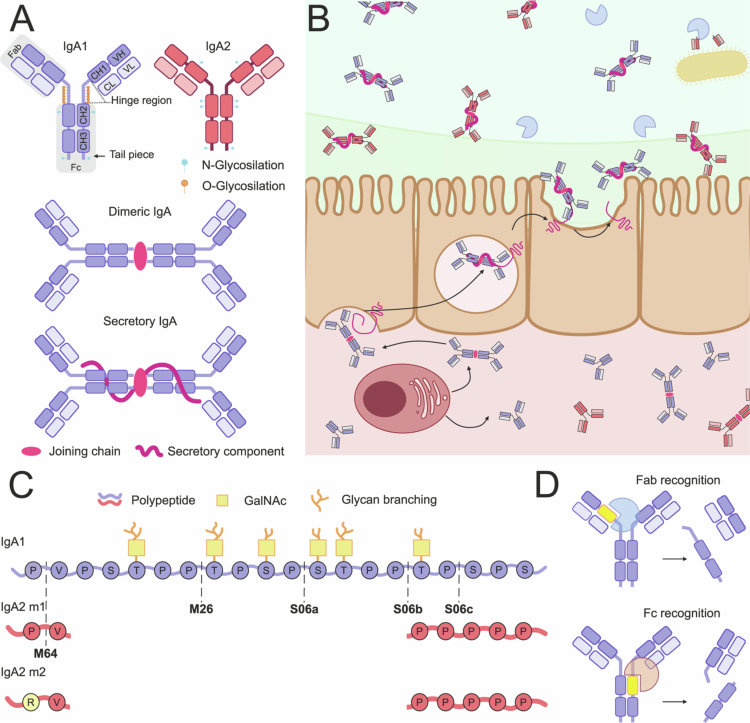
Immunoglobulin A proteoforms, secretion, and proteolytic dismantling. (A) Structural domain architecture of IgA1 and IgA2 subclasses. Overview of constant (CH, CL) and variable (VH, VL) domains, highlighting differences in hinge region length and both N- and O-glycosylation. Lower illustrations: dimeric IgA covalently linked via their C-terminal tailpieces and stabilized by the joining chain (pink) via disulfide bonds, and secretory IgA (sIgA) additionally wrapped by the secretory component (magenta) to confer enhanced protection against non-specific proteolysis. (B) Secretory pathway and mucosal role of sIgA. Production of monomeric and dimeric IgA by plasma cells within the lamina propria, followed by polymeric immunoglobulin receptor (pIgR)-mediated transcytosis across the epithelium to the apical gut lumen. Within the mucosa, sIgA mediates immune exclusion by cross-linking microbial antigens into large complexes to prevent epithelial adherence and invasion. However, resident and pathogenic bacterial peptidases can cleave the hinge region, separating the Fab arms from the Fc region to effectively neutralize antibody function and enable immune evasion. (C) Hinge region sequences and proteolytic susceptibility profiles. IgA1 features an extended, O-glycosylated hinge targeted by M64, M26, and S6 family proteases. Despite nine potential O-glycosylation sites (Ser/Thr), physiological proteoforms typically carry 3–6 glycans restricted to the six sites shown (T225, T228, S230, S232, T233, and T236).^237^ ^,^ ^238^ Notably, the S6 family includes members with distinct cleavage site specificities, depicted here as S6a, S6b, and S6c. In the IgA2 variants, a 13-amino acid deletion rigidifies the hinge region and eliminates the O-glycosylation sites and the cleavage motifs for M26 and S06. Crucially, allotype IgA2m(2) contains a Pro-to-Arg mutation conferring resistance to the M64 family.^239^ (D) Mechanisms of substrate recognition. Comparison between Fab-mediated (“top-down”; *e.g.*, M64 family) and Fc-driven (“bottom-up”; *e.g.*, M26 and S6 families) recognition required to precisely orient the catalytic site for IgA hinge cleavage.^191^ ^,^ ^240^ ^,^ ^241^

The intestinal mucosa is continuously monitored by the adaptive immune system, with sIgA serving as the dominant effector antibody (Figure 4A).^231^ Produced at gram quantities per day, sIgA permeates the mucus layer and coats the luminal surface, where it functions primarily through immune exclusion. In this process, sIgA binds microbial surface antigens such as adhesins or lipopolysaccharide, cross-linking bacteria into large immune complexes that become trapped within the mucus matrix and are removed by peristalsis and continuous mucus turnover (Figure 4B).^232^ ^,^ ^233^ Consequently, the ability to neutralize or evade sIgA represents a major selective advantage for mucosa-associated microbes. In a classic evolutionary arms race, bacteria have evolved specialized IgA-targeting mechanisms, including IgA-specific peptidases.^234^ Importantly, IgA cleavage can confer a communal benefit: by disrupting antibody-mediated agglutination, protease-producing species may reduce immune exclusion not only for themselves but also for neighboring non-proteolytic commensals, facilitating community persistence under immune pressure.^235^ ^,^ ^236^

#### 3.3.1. The target: structure and heterogeneity of secretory IgA

Mucosal IgA differs fundamentally from serum antibodies in both structure and function. Secretory IgA is predominantly dimeric, with two IgA monomers linked by a joining (J) chain and stabilized by the secretory component (SC), a remnant of the polymeric immunoglobulin receptor (pIgR) that protects IgA from nonspecific proteolysis.^242^ ^,^ ^243^ As in other immunoglobulin classes, antigen recognition is mediated by two Fab arms connected to the Fc region via a flexible hinge region (HR). Humans express two IgA subclasses distinguished primarily by hinge architecture (Figure 4C). IgA1, predominant in serum and the upper GI tract, contains an extended hinge enriched in proline, serine, and threonine residues and heavily decorated with O-linked glycans. This mucin-like domain confers flexibility and hydration but also creates a protease-susceptible site.^244^ ^,^ ^245^ In contrast, IgA2, which dominates in the distal intestine and colon, lacks the extended hinge insertion and O-glycans, rendering it intrinsically resistant to classical IgA1-specific proteases.^245^ Further complexity arises from IgA2 allotypic variation. The IgA2m(1) and IgA2m(2) allotypes differ by discrete hinge-proximal amino acid substitutions that critically influence susceptibility to bacterial proteases, underscoring how subtle host polymorphisms can shape microbial immune evasion strategies.^239^ ^,^ ^246^

#### 3.3.2. Enzymatic countermeasures: from isotype-specific to broad-spectrum cleavage

Proteolytic cleavage of IgA represents the most efficient bacterial strategy to defeat immune exclusion. IgA peptidases selectively target the hinge region, functionally decapitating the antibody. This cleavage abolishes agglutination by separating the Fab arms from the Fc region and prevents Fc-dependent engagement of the cognate transmembrane receptor FcαRI on phagocytes.^165^ ^,^ ^235^ Rather than forming a single protein family, bacterial IgA-neutralizing factors comprise three distinct mechanistic strategies, defined by how they engage and disable the antibody.

**The Gut-Specific “Pan-IgA” Strategy (M64 family).** The predominance of the protease-resistant IgA2 subclass in the distal intestine was long considered a host adaptation to the protease-rich environment of the gut.^245^ This assumption has been overturned by the discovery of a gut-specific IgA peptidase (IgAse) produced by the commensal-pathobiont *Thomasclavelia ramosa* (formerly *Clostridium ramosum*), which we highlight here as a representative mechanistic example of broad-spectrum immune evasion in the distal gut. This enzyme belongs to the M64 family and,^188^ in contrast to classical IgA peptidases that are restricted to IgA1, exhibits the capacity to cleave both IgA1 and the presumed protease-resistant IgA2 subclass. ^247^ ^,^ ^248^ Recent structural and biochemical analyzes have elucidated the molecular basis of this expanded specificity.^191^ ^,^ ^240^ ^,^ ^249^ Rather than docking onto the Fc region, as observed for other IgA-cleaving proteases, IgAse engages the antibody via a distinct “top-down” recognition mechanism (Figure 4D), binding the Fab portion, specifically the Cα1 domain adjacent to the hinge, to precisely position its catalytic site over the cleavage region.^191^ ^,^ ^240^ This orientation enables access to the sterically constrained IgA2 hinge and underlies the enzyme’s pan-IgA activity. Importantly, IgAse specificity is dictated by hinge-sequence composition rather than subclass identity per se. The enzyme efficiently cleaves the Pro–Val peptide bond present in both IgA1 and the IgA2m(1) allotype (Figure 4C), but fails to cleave the Pro–Arg variant found in the IgA2m(2) and IgA2(*n*) allotypes, in which the P1′ valine is replaced by arginine.^239^ ^,^ ^246^ This sharp allotype-dependent resistance highlights how subtle host polymorphisms can critically shape the success of bacterial immune-evasion strategies and exemplifies a finely tuned host–microbe evolutionary arms race.^235^ ^,^ ^250^

**The Respiratory/Classical IgA1-Specific Strategy (S06 and M26 families):** Historically, IgA peptidases were defined by their ability to cleave the extended hinge region of IgA1 while leaving IgA2 intact, a specificity that reflects both substrate accessibility and hinge architecture.^245^ These enzymes are predominantly associated with respiratory pathogens, including *Neisseria gonorrhoeae*, *Neisseria meningitidis*, *Haemophilus influenzae*, and *Streptococcus pneumoniae*, although homologous activities have also been described in gut-associated pathobionts such as *Streptococcus gallolyticus* and selected *E. coli* lineages.^235^ ^,^ ^251^ ^,^ ^252^ In contrast to M64 IgAse, classical IgA1 proteases engage the antibody primarily via Fc-proximal interactions and cleave the hinge region from a “bottom-up” orientation relative to the Fc (Figure 4D).^239^ ^,^ ^241^ The classical IgA1 proteases can be divided into two mechanistically distinct enzyme groups that nevertheless converge on the same functional outcome. Serine IgA1 proteases, exemplified by enzymes produced by *Neisseria* and *Haemophilus* species (MEROPS family S6), are secreted virulence factors that specifically recognize and cleave the IgA1 hinge region. Although these proteases preferentially target the O-glycosylated hinge of IgA1, experimental evidence indicates that substrate recognition is primarily driven by the peptide sequence rather than glycan decoration, as efficient cleavage is retained following deglycosylation of IgA1 or when short hinge-derived peptide substrates are used.^239^ ^,^ ^251^ Notably, IgA1 proteases display heterogeneity in their cleavage specificity within the hinge region. Different bacterial strains express enzymes that preferentially cleave distinct peptide bonds, most commonly Pro–Ser or Pro–Thr motifs, generating characteristic “cleavage types.” Variation in cleavage site selection reflects adaptation to subtle differences in hinge sequence composition and may contribute to differential susceptibility among host IgA1 variants, although the precise evolutionary advantage of maintaining multiple cleavage specificities remains incompletely understood.^239^ ^,^ ^251^ Converging on the same IgA1 target through a distinct catalytic mechanism are zinc-dependent IgA1 metalloproteases (family M26), exemplified by the pneumococcal IgA1 protease encoded by the *iga* gene of *Streptococcus pneumoniae*.^253^ Despite lacking homology to serine IgA1 proteases, this enzyme achieves the same functional outcome, precise separation of the Fab arms from the Fc region of IgA1, thereby abrogating immune exclusion and facilitating mucosal colonization.^253^ Functional studies further demonstrate that pneumococcal IgA1 protease activity directly undermines IgA1-mediated protection *in vivo*, underscoring the biological significance of IgA1-specific cleavage for immune evasion.^254^

Beyond these well-characterized systems, IgA1-cleaving activity has also been described in additional anaerobic and oral-associated pathobionts, although the underlying enzymatic machinery is less well defined. For example, *Prevotella melaninogenica* has been reported to express a cysteine peptidase (family C1) capable of cleaving IgA1 at a distinct site near the N-terminal region of the hinge, targeting peptide bonds different from those preferred by neisserial or streptococcal enzymes.^255^ ^,^ ^256^ These observations suggest that IgA1 cleavage has arisen multiple times independently, highlighting the strong and recurrent selective pressure exerted by IgA-mediated immune exclusion at mucosal surfaces.^239^ ^,^ ^251^

**Non-Proteolytic Evasion Strategies:** While pathogens colonizing the respiratory and GI tracts classically rely on peptidases to irreversibly cleave IgA,^235^ immune evasion is not limited to proteolysis. As an alternative to catalytic destruction, some pathogens employ non-enzymatic sequestration strategies to functionally neutralize the antibody. For example, *S. aureus*—noted for its proteases—also secretes staphylococcal superantigen-like protein 7 (SSL7).^257^ ^,^ ^258^ This protein binds the Fc region of IgA with nanomolar affinity, specifically targeting the Cα2-Cα3 interface.^258^ By occupying this site, SSL7 sterically hinders interaction with FcαRI and simultaneously binds complement component C5, thereby preventing its cleavage into C5a and C5b and blocking downstream membrane attack complex formation.^257^ ^,^ ^259^ Consequently, this dual-blockade inhibits Fc-mediated phagocytosis and immune effector functions, shielding the bacterium from clearance.^260^ However, a key distinction remains: compared to the catalytic and irreversible dismantling catalyzed by IgA peptidases, these binding strategies represent a stoichiometric and potentially reversible solution to the problem of immune exclusion.

### 3.4. Broad-spectrum assault: the oral-gut axis and gingipains

The connection between oral health and GI pathology is defined by the so-called “oral–gut axis”.^261^ Patients with severe periodontitis swallow large quantities of oral pathobionts, most notably *Porphyromonas gingivalis*, a “keystone oral pathogen”,^262^ and *Fusobacterium nucleatum*. While the acidic environment of the stomach typically acts as a barrier to oral microbes, conditions of hypochlorhydria, which are commonly associated with aging or the use of proton-pump inhibitors such as omeprazole and pantoprazole, permit survival during gastric transit and ectopic colonization of the lower GI tract.^263^ Consequently, although rare in young, healthy individuals, oral-to-gut translocation represents a significant risk factor in immunologically or physiologically compromised populations. Upon successful colonization, *P. gingivalis* acts as an illustrative prototype for oral-gut pathobionts, deploying a specialized repertoire of enzymatic effectors that fundamentally reshapes the gut immune microenvironment.^264^

#### 3.4.1. The enzymatic arsenal: gingipains

The virulence of *P. gingivalis* is largely driven by potent cysteine proteases termed gingipains,^265^ named after both the pathogen species and their structural relationship to papain-like proteases. These enzymes dominate the bacterial secretome, accounting for approximately 85% of the organism’s total extracellular proteolytic activity.^266^ ^,^ ^267^ Gingipains belong to the C25 family and are classified according to strict substrate specificity: RgpA and RgpB (gingipain R) cleave peptide bonds C-terminal to arginine (R) residues, whereas Kgp (gingipain K) cleaves after lysine (K) residues. Structurally, these proteases frequently function as multifunctional complexes; while RgpB consists primarily of a catalytic domain, RgpA and Kgp possess large C-terminal hemagglutinin/adhesin domains that mediate attachment to host cells and co-aggregating bacteria, thereby concentrating proteolytic activity at the cell surface.^265^ ^,^ ^266^ In contrast to the highly specific, junction-targeted activity of BFT, gingipains operate as molecular bulldozers with broad substrate specificity, functionally resembling the digestive protease trypsin. However, unlike trypsin, which is spatially confined to the intestinal lumen, gingipains target a wide spectrum of host immune, signaling, and structural proteins within tissues and at mucosal interfaces, enabling extensive immune subversion and tissue destruction.^268^ ^,^ ^269^

#### 3.4.2. Immune dysregulation and complement subversion

A primary function of gingipains is the systematic dismantling of host innate immune defenses.^262^ ^,^ ^266^ These proteases efficiently degrade inflammatory cytokines such as IL-8 and IL-1β, thereby disrupting chemotactic gradients required for neutrophil recruitment, the primary phagocytic cell type responsible for bacterial clearance, to sites of infection.^270^ In parallel, gingipains manipulate the complement system, a central arm of innate immunity. Both Rgp and Kgp cleave C3, the pivotal complement component responsible for opsonization, thereby impairing phagocytosis by macrophages and neutrophils.^271^ Paradoxically, gingipains also generate biologically active C5a fragments. Rather than promoting bacterial clearance, this selective C5a generation drives maladaptive crosstalk between the C5a receptor (C5aR1) and Toll-like receptor 2 (TLR2) on neutrophils.^62^ This signaling axis uncouples inflammatory cytokine production from effective antimicrobial killing, sustaining a state of chronic, low-grade inflammation that supplies proteinaceous nutrients through inflammatory exudates and heme release while preventing pathogen eradication. This immune manipulation exemplifies the “inflammophilic” lifestyle of *P. gingivalis*. Consistent with its designation as a keystone pathogen,^262^ *P. gingivalis* exerts disproportionate effects on the microbial ecosystem despite low abundance. By impairing neutrophil and complement function, it precipitates a transition from eubiosis to dysbiosis, fostering the expansion of a polymicrobial, pro-inflammatory community that perpetuates mucosal damage.^262^ ^,^ ^272^

#### 3.4.3. Signaling hijacking: activation of protease-activated receptors (PARs)

Beyond direct degradation, gingipains function as potent signaling effectors through activation of PARs, particularly PAR1, PAR2, and PAR4.^273^ ^,^ ^274^ These G-protein-coupled receptors are expressed on both epithelial and immune cells and are uniquely activated by site-specific proteolytic cleavage of their extracellular N-termini, which exposes an intramolecular tethered ligand that functions as a self-agonist. In the intestinal context, gingipain-mediated activation of PAR2 induces the production of pro-inflammatory cytokines, notably IL-6 and TNF-*α*, and stimulates the release of matrix metalloproteinases such as MMP-9 from epithelial cells and neutrophils.^273^ ^,^ ^274^ This establishes a synergistic destructive loop: gingipain-induced PAR2 signaling promotes secretion of host pro-MMPs, which are subsequently processed into active enzymes by gingipains themselves, accelerating extracellular matrix degradation, facilitating bacterial invasion, and liberating nutrients including peptides and heme. Importantly, oral-to-gut translocation of *P. gingivalis* links oral dysbiosis to exacerbation of colitis and underscores the systemic relevance of periodontal disease.^275^ Chronic gingipain-driven inflammation may further facilitate colorectal carcinogenesis, in part by enabling colonization by oncogenic species such as *F. nucleatum*.^276^

#### 3.4.4. Indirect tissue destruction: disarming the antiprotease shield

In addition to direct proteolysis, *P. gingivalis* promotes tissue destruction through targeted inactivation of host antiprotease defenses.^266^ ^,^ ^277^ Under physiological conditions, mucosal tissues are protected from inadvertent damage by neutrophil-derived serine proteases, particularly neutrophil elastase and proteinase 3, two serine proteases of the S1 family, via secretory inhibitors such as secretory leukocyte protease inhibitor (SLPI) and elafin.^278^ Gingipains efficiently degrade both SLPI and elafin, abolishing this critical regulatory checkpoint.^277^ ^,^ ^279^ ^,^ ^280^ Loss of antiprotease control unleashes unopposed host protease activity, creating a destructive feed-forward loop in which recruited neutrophils release elastase that remains active due to gingipain-mediated inhibitor degradation.^279^ ^,^ ^280^ This proteolytic “friendly fire” accelerates barrier breakdown, tissue injury, and nutrient release while simultaneously undermining physical containment of the pathogen. By dismantling the host's own antiprotease shield, *P. gingivalis* effectively co-opts host neutrophils to amplify tissue damage, turning the immune response into a primary driver of disease pathology.

## 4. Conclusion and future perspectives

Microbial peptidases extend far beyond their classical role in passive protein degradation, acting as central regulators of gut ecosystem structure, interspecies communication, and host–microbe interactions. Across ecological and pathogenic contexts, proteolysis repeatedly appears as a decisive, typically irreversible mechanism capable of reshaping biological systems, from remodeling biofilm architecture and rewiring QS networks, to breaching mucosal and epithelial barriers, modulating immune surveillance, and activating host signaling pathways. In contrast to many microbial metabolites, whose effects are transient and reversible, protease-mediated cleavage events frequently function as molecular switches, permanently altering substrate function and biological outcome. This positions microbial peptidases as particularly powerful effectors at the interface between eubiosis and dysbiosis.^31^

Crucially, the biological impact of proteolysis is governed less by gene abundance than by spatiotemporally regulated enzymatic activity. Genomic and metagenomic surveys alone are therefore insufficient to capture the functional proteolytic landscape of the gut. Instead, approaches that directly resolve enzyme activity, such as functional metaproteomics, positional proteomics, and activity-based protein profiling, are critical for distinguishing dormant, inhibited, or mislocalized proteases from those actively shaping host and microbial biology.^27^ ^,^ ^28^ This functional lens exposes proteolytic activities with sensory or immunomodulatory roles that are invisible to sequence-based analyzes.^31^ ^,^ ^61^

Collectively, these observations set the stage for several near-term priorities for the field. First, improved spatial resolution will be required to define where proteolytic events occur within the GI tract, including the lumen, mucus layer, biofilm matrix, and epithelial interface, and how this localization determines biological outcome. Second, distinguishing the contributions of resident, transient, and opportunistic taxa, across commensal, pathobiont, and pathogenic lineages, will be essential for interpreting causality in dysbiosis-associated disease, particularly given that proteolytic function can shift between beneficial, neutral, or deleterious roles depending on ecological and host context. Third, while microbial proteases represent attractive conceptual targets for therapeutic modulation, most current strategies remain at an early stage and require validation in physiologically relevant *in vivo* gut models before translational claims can be justified.^143^ ^,^ ^155^ Together, these considerations position microbial proteolysis not as a peripheral feature of the microbiome, but as a core regulatory layer whose systematic interrogation will be essential for understanding, and ultimately improving, gut ecosystem function and host health.
